# Simulating the Oceanic Migration of Silver Japanese Eels

**DOI:** 10.1371/journal.pone.0150187

**Published:** 2016-03-16

**Authors:** Yu-Lin Chang, Yasumasa Miyazawa, Mélanie Béguer-Pon

**Affiliations:** 1 Institute of Marine Environmental Sciences and Technology, National Taiwan Normal University, Taipei, Taiwan, 11677; 2 Application Laboratory, Japan Agency for Marine-Earth Science and Technology, Yokohama, Japan, 236–0001; 3 Department of Oceanography, Dalhousie University, Halifax, Canada, B3H 4R2; Institute of Marine Research, NORWAY

## Abstract

The oceanic migration of silver Japanese eels starts from their continental growth habitats in East Asia and ends at the spawning area near the West Mariana Ridge seamount chain. However, the actual migration routes remain unknown. In this study, we examined the possible oceanic migration routes and strategies of silver Japanese eels using a particle tracking method in which virtual eels (v-eels) were programmed to move vertically and horizontally in an ocean circulation model (Japan Coastal Ocean Predictability Experiment 2, JCOPE2). Four horizontal swimming strategies were tested: random heading, true navigation (readjusted heading), orientation toward the spawning area (fixed heading), and swimming against the Kuroshio. We found that all strategies, except random swimming, allowed v-eels swimming at 0.65 m s^−1^ to reach the spawning area within eight months after their departure from the south coast of Japan (end of the spawning season). The estimated minimum swimming speed required to reach the area spawning within eight months was 0.1 m s^−1^ for true navigation, 0.12 m s^−1^ for constant compass heading, and 0.35 m s^−1^ for swimming against the Kuroshio. The lowest swimming speed estimated from tracked Japanese eels at sea was 0.03 m.s^−1^, which would not allow them to reach the spawning area within eight months, through any of the tested orientation strategies. Our numerical experiments also showed that ocean circulation significantly affected the migration of Japanese v-eels. A strong Kuroshio could advect v-eels further eastward. In addition, western Pacific ocean currents accelerated the migration of navigating v-eels. The migration duration was shortened in years with a stronger southward flow, contributed by a stronger recirculation south of Japan, an enhanced subtropical gyre, or a higher southward Kuroshio velocity.

## Introduction

Anguillid eels are widely distributed worldwide in various habitats such as rivers, lakes, estuaries, and coastal marine waters[1]. Eels are an important source of food for sea animals and birds and have a high economical value [2]. Therefore, they are of great interest in oceanography, meteorology, and biology [3]. The Japanese eel *Anguilla japonica* is mainly distributed in the western Pacific Ocean [2]. Its abundance declined in the past three decades, and it is now listed as an endangered species in the IUCN red list [2]. Overfishing, turbine passage mortality, and habitat degradation are among the main factors responsible for this decline [4]. Changes in oceanic conditions may also significantly affect eel recruitment [5–7]. The biology of Japanese eels, particularly the oceanic migration from continental waters to the spawning grounds, is not well understood because of a lack of direct observations [8].

**Fig 1:**
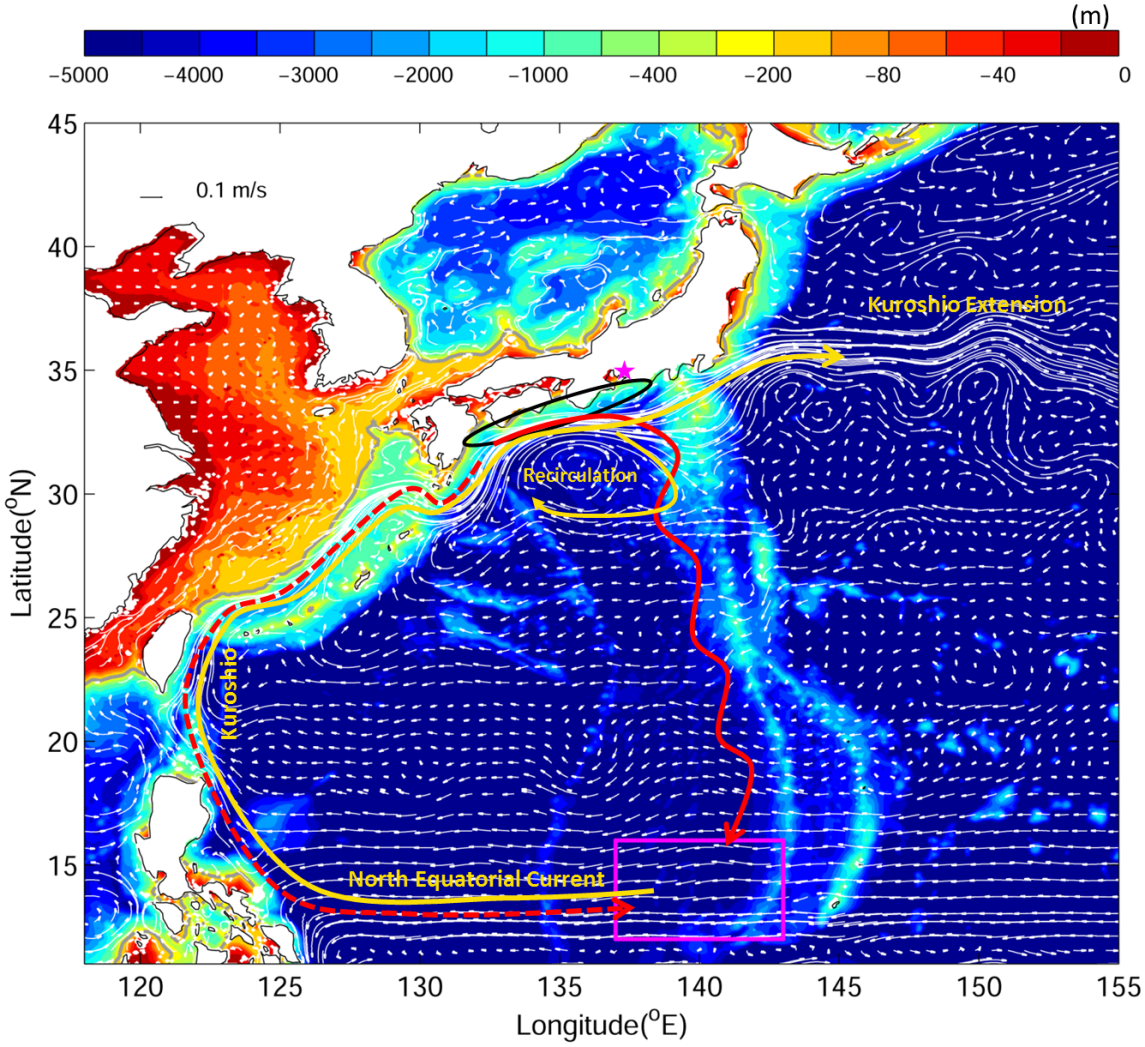
Major bathymetric (color) features of the study area in the western North Pacific Ocean. Solid white curves with arrows indicate major ocean currents in top 300 m average. The magenta box represents the spawning area. The black ellipse marks the v-eel release area. The two red curves are the hypothesized seaward migration routes from (solid) Tsukamoto[15] and (dash) Yokose [16]. The star is located next to Mikawa Bay. The yellow curves identify the main ocean currents.

The early phase of the seaward migration of Japanese silver eels was observed in Mikawa Bay, south of Japan (Fig 1) [9]. During a three-year observation period (1997–2000), the departure of silver Japanese eels from continental waters was recorded in late autumn to winter and was found to be the greatest from November to January. The spawning area of Japanese eels is located west of Mariana Islands [10,11] (Fig 1), approximately 2,300 km from Japan. Based on the larval distribution and abundance observed over the past 52 years (1956–2007), Shinoda et al. [12] suggested that the spawning season begins in April and ends in August. The long-distance migration of adult eels has not yet been fully observed. Thus, knowledge of the migratory path, navigation/orientation cues that eels use, and environmental conditions that eels experience is very limited. Filling this knowledge gap is crucial for research and management purposes.

The Kuroshio, the recirculation south of Japan, and the subtropical gyre (south of the Kuroshio and north of the North Equatorial Current) are the main ocean features that eels encounter during their migratory route from continental waters to the spawning area (Fig 1). The Kuroshio is the western boundary current in the North Pacific Ocean, with a mean transport of 42 Sv in the upper 1000 m (1 Sv = 10^6^ m^3^ s^−1^) and a surface speed of 1–2 m s^−1^ south of Japan [13]. The recirculation south of Japan is a local anticyclonic gyre. The diameter of the recirculation is approximately 500 km, and its size and strength vary with time [14].

Several hypotheses related to the oceanic migration routes of Japanese silver eels have been formulated. One suggested an “along-Kuroshio route” [15] and another suggested an “anti-Kuroshio route” [16] (Fig 1, red curves). Both hypotheses suggested surface migration [i.e., without diel vertical migration (DVM)]. In the case of the along-Kuroshio route hypothesis, the silver eels would be entrained in the Kuroshio south of Japan and drift eastward because of the strong Kuroshio. When the silver eels meet the recirculation gyre, they would move southward and head to the spawning area using environmental cues such as the geomagnetic field, either orientating using one of its component (intensity or declination) or truly navigating using a bi-directional map based on two components of the geomagnetic field (inclination and total intensity of inclination or declination) [17]. In the case of the anti-Kuroshio route hypothesis, the silver eels would migrate against the Kuroshio and the North Equatorial Current further upstream. Eels are able to swim against a current of 1–2 m s^−1^ in laboratory experiments [18]. Although swimming against the current is highly energy consuming, the silver eels would be able to retrace their larval migration route, for instance, by following some olfactory attractants [16], or eels may smell each other.

In previous studies, it was assumed that silver eels constantly swim near the surface. However, DVMs have recently been observed in the Japanese silver eel [19,20] as well as in other anguillid species [21,22]. Using pop-up satellite archival tags to track silver eels south of Japan, Manabe et al. [19] observed that eels swam at depths between 100 and 500 m at nighttime and between 500 and 800 m at daytime. Similar observations on swimming depths have been obtained from another study, which tracked acoustically tagged Japanese eels (i.e., using a small internal device causing no drag) in the same area [20]. Manabe et al. [19] also evaluated the horizontal swimming speed (excluding the background current speed) of tracked Japanese silver eels during a 1400-km migration near Japan, finding values between 2.2 and 15.1 km day^−1^ (0.03–0.17 m s^−1^).

In the present study, the possible oceanic migration routes and strategies of Japanese silver eels in the western Pacific Ocean were simulated. A three-dimensional (3D) particle tracking method was used to simulate the movement of virtual eels (v-eels) with various swimming behaviors in a physical environment. The first objective was to assess which behaviors (heading and swimming speeds) allow v-eels to reach the spawning area in time for breeding. The minimum swimming speeds required to reach the spawning area in time were assessed. The second objective was to investigate the effect of extreme ocean circulation conditions on the migration of v-eels.

## Data and Methods

An individual-based model was used in this study to simulate biological processes at the level of individuals or small groups of individuals in the population [23]. The movements of particles in the individual-based model were carried by ocean currents with the inclusion of biological behaviors such as swimming direction, horizontal swimming, and DVM.

### Simulation of ocean circulation

The currents and hydrological fields used in particle tracking were extracted from the ocean circulation reanalysis of the Japan Coastal Ocean Predictability Experiment 2 (JCOPE2) [24]. JCOPE2 used a data-assimilated ocean model constructed from the Princeton Ocean Model (POM) with a generalized coordinate system [25]. The JCOPE2 model domain encompasses the western North Pacific (10.5–62°N, 108–180°E), with a horizontal resolution of 1/12° (8–9 km) and 46 vertical layers. The model external forcing includes wind stresses and net heat/freshwater fluxes at the sea surface converted from the six-hourly atmospheric reanalysis produced by the *National Centers for Environmental Prediction/*National Center for Atmospheric Research. Satellite and in situ temperature and salinity data were assimilated into the model. Daily JCOPE2 reanalysis fields cover January 1993 to the present.

A comparison between the simulated trajectories of passive particles carried by the JCOPE2 and the observed trajectories was shown in a previous study [26]. The JCOPE2 currents were also validated against drifter-derived velocities. JCOPE2 reanalysis reproduced the general circulation in the western Pacific Ocean well, particularly the path and strength of the Kuroshio.

### Particle-tracking scheme

#### Particle movement caused by ocean currents

In this study, we used the 3D particle-tracking scheme developed by Ohashi and Sheng [27], which is based on the fourth-order Runge–Kutta method [28]. The position of a particle is tracked from its position at time *t (${\vec{x}}^{t}$)* to a new position at time *t +* Δ*t (${\vec{x}}^{t+\Delta t}$)* based on

$${\vec{x}}^{t+\Delta t}={\vec{x}}^{t}+\int_{t}^{t+\Delta t}\vec{u}(\vec{x},t)dt+\vec{\delta }$$ (1)

where $\vec{u}$ is the ocean current from the JCOPE2 reanalysis and $\vec{\delta }$ represents the additional displacement associated with a random walk during this time interval, representing unresolved sub-grid turbulent flow and other local processes [29]. The estimated δ(x,y) was approximately 600 m, and δ(z) was approximately 20 m. Note that the vertical velocity was not used in simulation. The integration time step was three hours. Bilinear interpolation was used to establish a continuous velocity dataset in time and space. The same tracking scheme was used by Chang et al. [26] to investigate migration of Japanese eel larvae in the western Pacific Ocean, and it was also applied to the simulation of the long-distance migration of adult eels in the Sargasso Sea [30].

#### Particle movement induced by active swimming

Various vertical and horizontal swimming behaviors of Japanese eels were considered in this study, including swimming direction, speed, and depth.

Swimming courses were selected on the basis of hypothetical migration routes. Four horizontal behaviors were simulated: random swimming, compass orientation, true navigation, and swimming against the Kuroshio. In the case of random swimming, the v-eels do not have a goal destination and swim in a random direction at each time step. Rypina et al. [31] showed that the time step could have a significant effect on the dispersal of randomly swimming particles. Two values were tested in addition to the three-hourly one in the case of the randomly swimming scenario: 6 and 12 hours. In the case of compass orientation, the v-eels were programmed to constantly swim in the same direction determined at their departure, i.e., southeastward (150–165 degree clockwise from true north, depending on the release location). In the absence of ocean circulation, this heading would lead the v-eels to the spawning area. In the case of true navigation, the v-eels know their exact location at each time step as well as the location of the spawning area, which they were programmed to swim toward. If the migratory paths were altered by background currents, the v-eels could adjust their swimming direction. In the case of swimming against the current, the v-eels were programmed to swim along a shoreward migration route according to the mean Kuroshio and North Equatorial Current paths; they first swam against the Kuroshio and then against the North Equatorial Current.

The horizontal swimming speed of Japanese silver eels tracked using pop-up satellite archival tags was estimated at approximately 0.03–0.17 m s^−1^ [19]; their optimum swimming speed is unknown. However, laboratory experiments performed by Palstra et al. [32] on European silver eels suggested an optimal swimming speed between 0.61 and 0.68 m s^−1^. To conduct our numerical experiments, we decided to use this range of reported horizontal swimming speeds (0.05 to 0.65 m s^−1^) to simulate and examine the migration of v-eels.

The observed DVM of Japanese silver eels indicated that they swim at depths between 100 m and 300 m at nighttime and between 500 m and 800 m at daytime [19]. In our numerical experiments, v-eels were set to swim at a constant depth of 200 m at nighttime and instantly moved to a depth of 600 m at daytime. In preliminary experiments, no significant difference in the migration duration of v-eels swimming at 600 m and those swimming at 800 m at daytime was observed. Similarly, the results were not sensitive to nighttime diving depth at 200 m or between 100 to 300 m. Therefore, the experiments conducted at 600 m at daytime were presented. The duration of day and night was determined by sunrise and sunset each day, and sunrise and sunset were set seasonally. The day length for spring and autumn was set to 12 hours (6 am to 6 pm) and was shortened to 10 hours (7 am to 5 pm) for winter (December–February). A longer day length of 14 hours (5 am to 7 pm) was set for summer (June–August).

### Experimental design

Numerical experiments were conducted to examine the oceanic migration of v-eels under different strategies. The v-eel release region and period were selected on the basis of the observations. In each experiment, particles were released near the southern coast of Japan (Fig 1; 132–138°E, 32–35°N, with an interval of 10 km). The downstream migration of silver eels in Japanese rivers was observed to reach a peak in December [9]. Because the v-eels in our numerical experiments were released in near-shore regions rather than rivers, the initial release time along the southern coast of Japan began later, i.e., from January 1 to February 28, and be staggered by a time interval of 5 days within this two-month period. Overall, 4944 v-eels (412 per run, 12 runs per experiment) were released in each experiment. Japanese eel larvae were observed to hatch in the spawning area in late spring and summer [12]. The migration period estimated from observations was approximately four to eight months. We therefore set the tracking duration of our numerical experiments to eight months.

**Fig 2:**
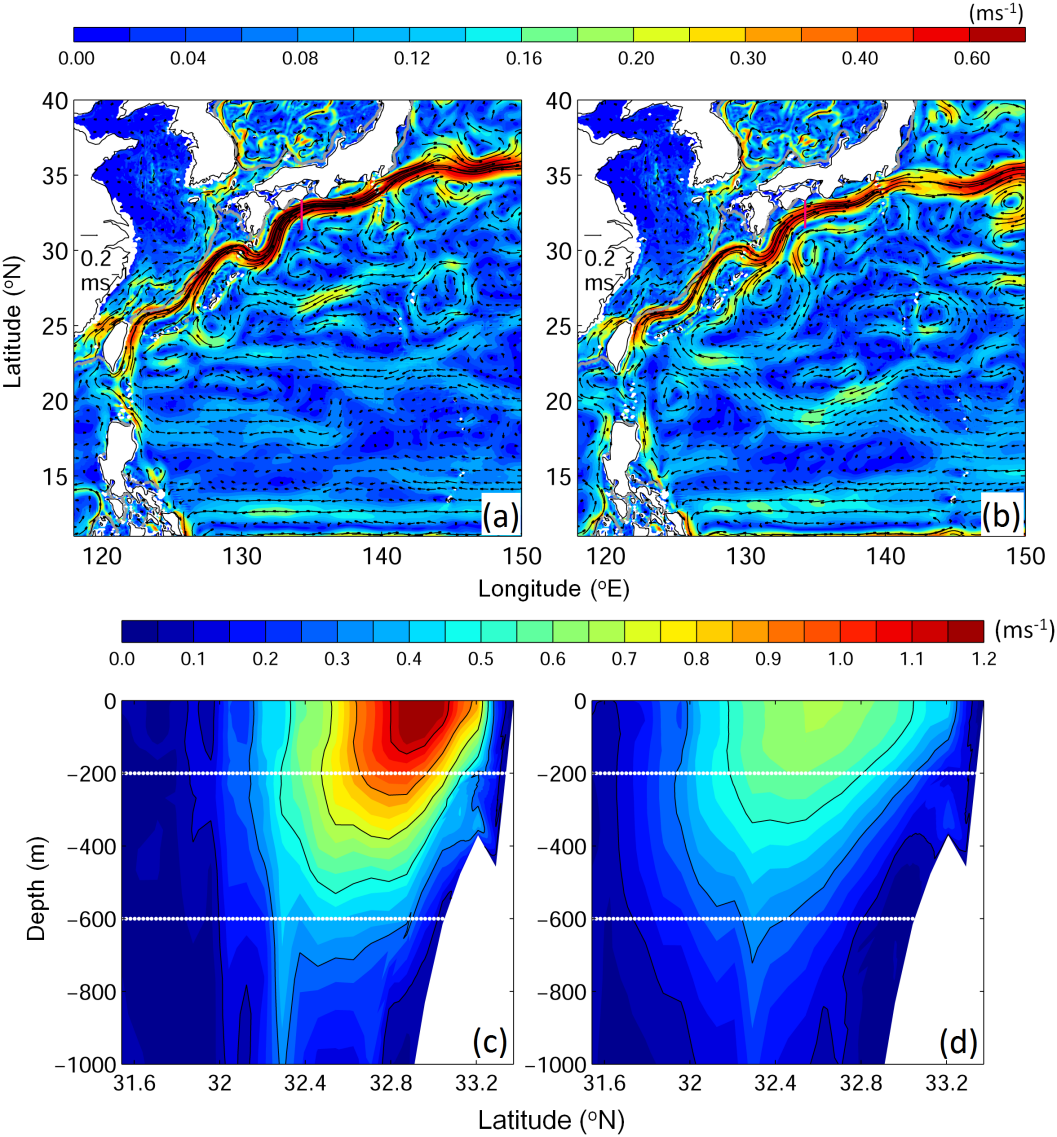
(a)(b)Top 600 m mean flow field and (c)(d) vertical profile of ocean velocity at 134.2E [magenta lines in (a)(b)] show the cases of (a)(c) a strong Kuroshio and (b)(d) a weak Kuroshio. White lines show the reference v-eel migration depths.

The numerical experiments conducted in this study are summarized in Table 1. In the first group of experiments (Exps. 1–4), the horizontal swimming behaviors were compared. In the second group of experiments (Exps. 5–8), the effect of swimming speed on migration was examined. Furthermore, all experiments were conducted under two extreme oceanic conditions, a strong Kuroshio (Exp. A, 48 Sv; 0–1000 m; Fig 2a & 2c) and a weak Kuroshio (Exp. B; 34 Sv; Fig 2b & 2d), to assess the influence of the Kuroshio on v-eel migration. The strong (or weak) Kuroshio case corresponded to the period when the Kuroshio remained stronger (or weaker) than average during v-eel migration through the Kuroshio (January to March). The ocean reanalysis from JCOPE2 in the year 2002 (strong) and 1995 (weak) were selected. An experiment without ocean circulation was also performed to assess the role of the ocean current in the case where v-eels were truly navigating toward the spawning area.

**Table 1:** Parameters used in the numerical experiments [note that in all experiments, v-eels are programmed to move vertically (DVM), with swimming at depths of 200 m at night and 600 m during the day].

Exp. Name	Swimming Speed (m s^−1^)	Swimming direction
Exp. 1	0.15	Random
Exp. 2	0.15	Compass
Exp. 3	0.15	True navigation
Exp. 4	0.15	Against the Kuroshio
Exp. 5	0.05	True navigation
Exp. 6	0.10	True navigation
Exp. 7	0.65	True navigation
Exp. 8	0.65	Against the Kuroshio

The minimum swimming speeds that would allow v-eels to arrive at the spawning area under different swimming behaviors were obtained by testing different swimming speeds. The swimming speed that leads to the mean migration duration close to (but less than) eight months (by the end of hatch season) was determined.

### Data analysis

A generalized linear model (GLM; binomial family) was used to evaluate the probability of success (proportion of v-eels reaching the spawning area) over time and according to the experiments (both in interaction). GLM showed that all experiments led to significantly different success rates (p < 0.001 for all combinations). Differences among experiments in the average migration duration were assessed using the Wilcoxon test. Differences in the success rate were assessed using the chi-square test.

## Results

**Fig 3:**
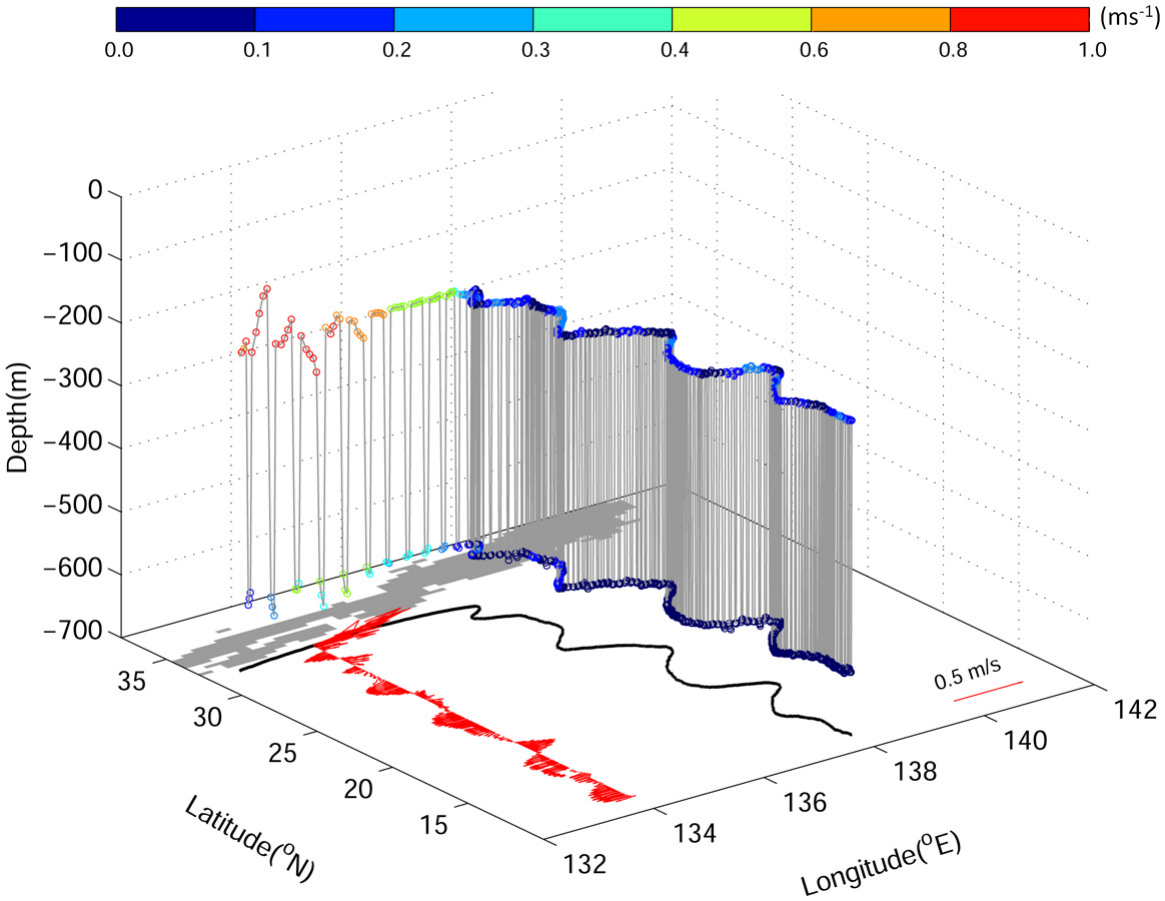
An example of the 3D trajectories of v-eel in Exp. 3A (true navigation, 0.15 m s^−1^). Colored dots are the ocean current speed. The black curve is the horizontal projection of the migration path. Red arrows are the along-path ocean velocities.

The 3-D trajectory of a randomly selected v-eel programmed to truly navigate from the coast of Japan to the spawning area was shown in Fig 3 as an example visualization result.

### Success of numerical experiments

#### Random swimming (Exp. 1)

**Fig 4:**
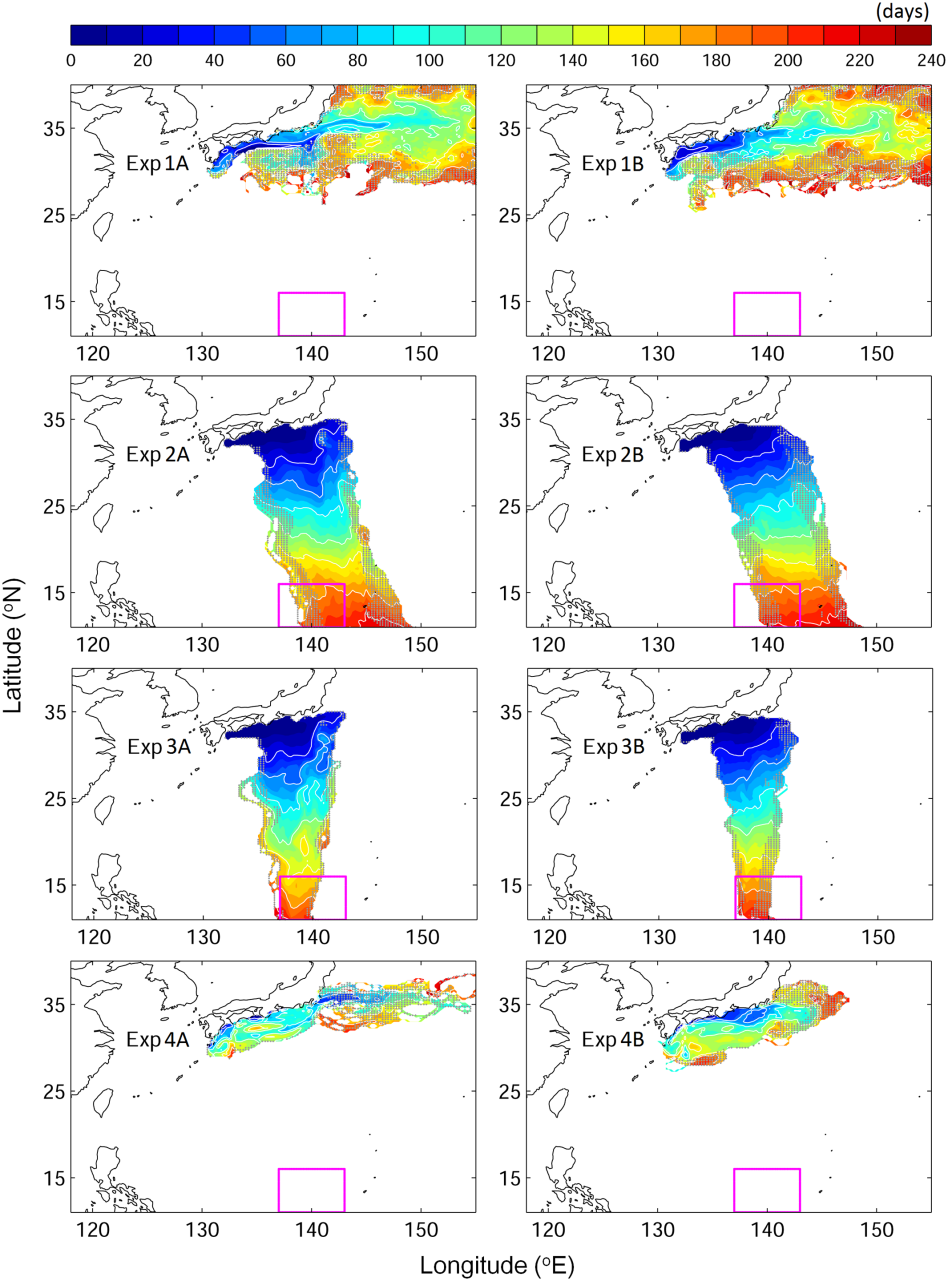
Time evolution (days) of the depth-integrated trajectories of v-eels for each tested scenario at swimming speed of 0.15 m s^−1^. Left panels are for cases A in which the Kuroshio is strong, and right panels are for cases B in which Kuroshio is weak. (from top to bottom) Exp. 1 (random), Exp. 2 (compass), Exp. 3 (true navigation), and Exp. 4 (against Kuroshio). Gray dots mark the locations where the number of v-eels is less than 1%. The magenta box shows the spawning area.

None of the v-eels swimming in a random direction at a speed of 0.15 or 0.65 m s^−1^ (Exp. 1, Table 1) succeeded in reaching the spawning area within eight months. V-eels in this experiment were widely distributed north of 25N, on the southern and eastern sides of Japan, at the end of the tracking period (Fig 4). In addition, some of the v-eels moved northward, which was the opposite direction of the spawning area. The time step of 6 and 12 hours increased the dispersal but still did not allow any v-eels to reach the spawning area on time.

#### Compass orientation (Exp. 2)

**Fig 5:**
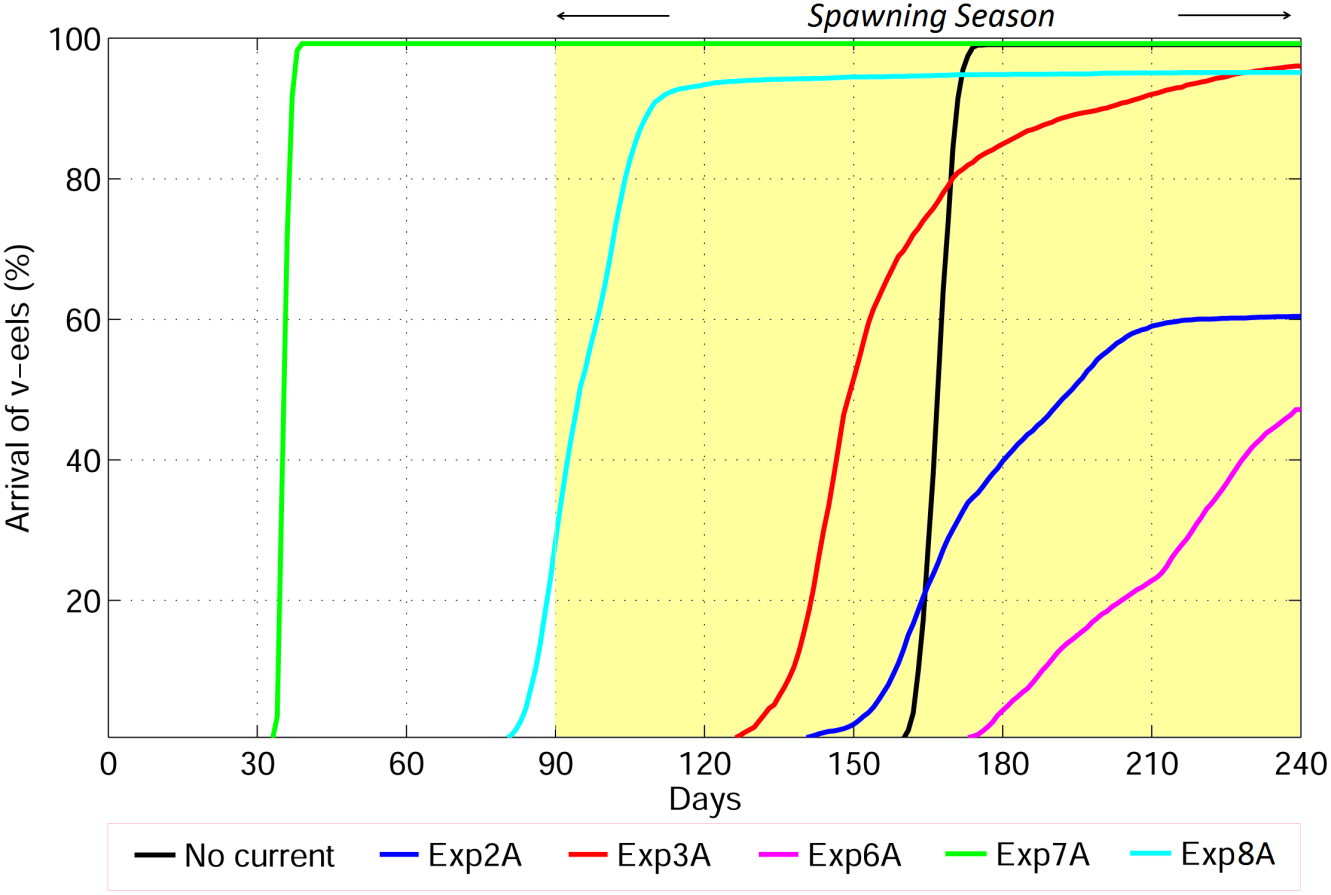
Percentage of v-eels arriving at the spawning area over time in Exp. 2A (compass orientation 0.15 m s^−1^), Exp. 3A (true navigation, 0.15 m s^−1^), Exp. 6A (0.1 m s^−1^), Exp. 7A (0.65 m s^−1^), and Exp. 8A (0.65 m s^−1^ against the Kuroshio). The yellow area marks the spawning period.

The migratory route of v-eels that swim in a fixed direction toward the spawning area (compass orientation) at 0.15 m s^−1^ was deflected eastward because of the Kuroshio. Therefore, 39.7% of these v-eels never entered the spawning area (Fig 4, Exp. 2, and Fig 5). Among the 60.3% v-eels that successfully entered the spawning area, the first v-eel arrived after 140 days of migration, and the mean migration duration for successful arrival was 174.4–175.1 days (Tables 2 and 3).

**Table 2:** Mean migration duration for successful v-eel arrival (days) from the south coast of Japan to the spawning area located 2300 km further south. Cases A and B are the strong and weak Kuroshio cases, respectively. Mean migration duration is significantly different between case A and case B for each experiment (Wilcoxon test, p < 0.001).

Exp. Name	Case A	Case B
Exp. 1	--	--
Exp. 2	174.4 ± 1.5	175.1 ± 1.3
Exp. 3	156.5 ± 1.5	167.1 ± 1.1
Exp. 4	--	--
Exp. 5	--	--
Exp. 6	224.3 ± 10.0	235.5 ± 9.7
Exp. 7	36.0 ± 0.2	36.4 ± 0.2
Exp. 8	98.9 ± 0.9	93.3 ± 0.9

**Table 3:** Mean arrival rate (%) for successful v-eel arrival from the south coast of Japan to the spawning area. Mean arrival rates are significantly different among experiments (chi-square test, p < 0.001).

Swimming Speed	Random Directionadd	Compass Orientation	True Navigation	Against Currents
0.05 m s^−1^	--	--	--	--
0.15 m s^−1^	--	60.3	96.1	--
0.65 m s^−1^	--	72.3	99.3	95.1

#### True navigation (Exps. 3, 5, 6, 7)

**Fig 6:**
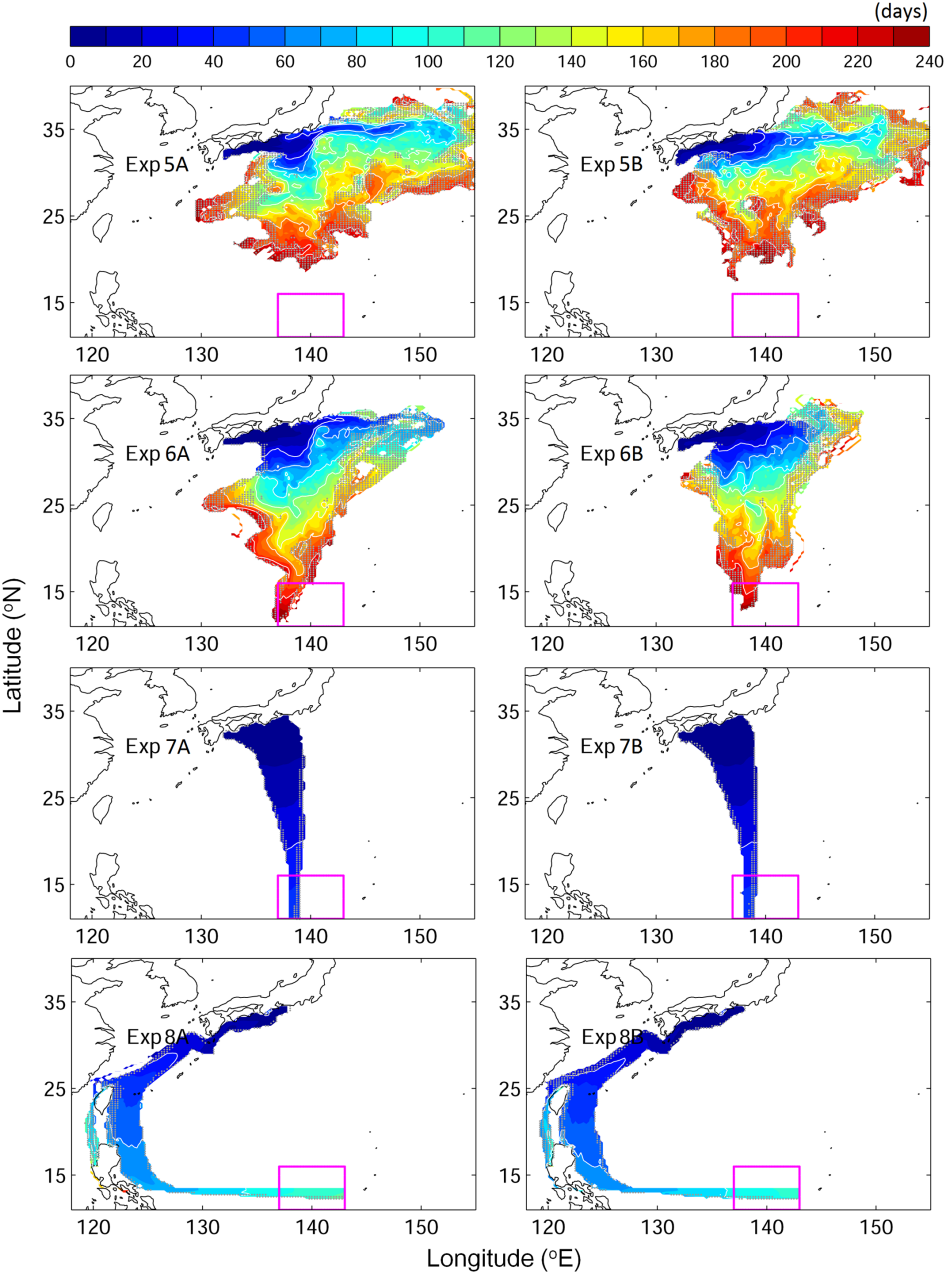
Same as Fig 2 but for v-eels swimming with true navigation at different swimming speeds: (from top to bottom) Exp. 5 (0.05 m s^−1^), Exp. 6 (0.1 m s^−1^), Exp. 7 (0.65 m s^−1^), and Exp. 8 (0.65 m s^−1^ against the Kuroshio).

None of the truly navigating v-eels swimming at 0.05 m s^−1^ reached the spawning area in eight months, i.e., before the end of the spawning period (Fig 6, Exp. 5). At this swimming speed, truly navigating v-eels were advected by the Kuroshio, moving eastward to the Kuroshio extension region. However, at a constant minimum swimming speed of 0.1 m s^−1^, truly navigating v-eels succeeded in crossing the Kuroshio and reached the spawning area on time (Fig 6, Exp. 6). The duration of migration and the related success rates were sensitive to the swimming speed (Fig 5). At the highest tested swimming speed, 0.65 m s^−1^, more than 98% of the v-eels reached the spawning area within 37 days, i.e., 53 days before the beginning of the spawning period (Figs 5 and 6, Tables 2 and 3). At a swimming speed of 0.1 m s^−1^ (Exp. 5), the first successful v-eels reached the spawning area 175 days after their departure, and the success rate reached 45.1% at the end of the tracking period (240 days). The mean migration duration for successful v-eels swimming at 0.1 m s^−1^ was 224.3–235.5 days. At a slightly higher swimming speed of 0.15 m s^−1^ (Exp. 3), v-eels started reaching the spawning area 130 days after their departure, and 94.3% of them reached it before the end of the spawning/tracking period. The mean migration duration was 156.5–167.1 days. (Figs 4 and 5, Table 2). A swimming speed of 0.15 m s^−1^ (Exp. 3) produced a high arrival rate, and the mean arrival time was near the middle of the spawning season.

#### Swimming against the Kuroshio (Exps. 4 and 8)

At a swimming speed of 0.15 m s^−1^, none of the v-eels that swam against the Kuroshio (Exp. 4) succeeded in reaching the spawning area (Fig 4, Exp. 4). At this speed, v-eels could not counteract the Kuroshio effect, the speed of which ranges from 0.2 to 1.0 m s^−1^ at 200- and 600-m depths, respectively. Most v-eels in this experiment were trapped in south Japan, and some of them were carried northeastward. V-eels swimming against the Kuroshio at a speed of 0.65 m s^−1^ were able to move toward the spawning area (Fig 6, Exp. 8), and 90% of them reached it within four months, i.e., in the early spawning season (Fig 5). A minimum swimming speed of 0.35 m s^−1^ was required for v-eels swimming against the Kuroshio to reach the spawning area by the end of the hatching season.

### Effect of the Kuroshio and ocean currents

**Fig 7:**
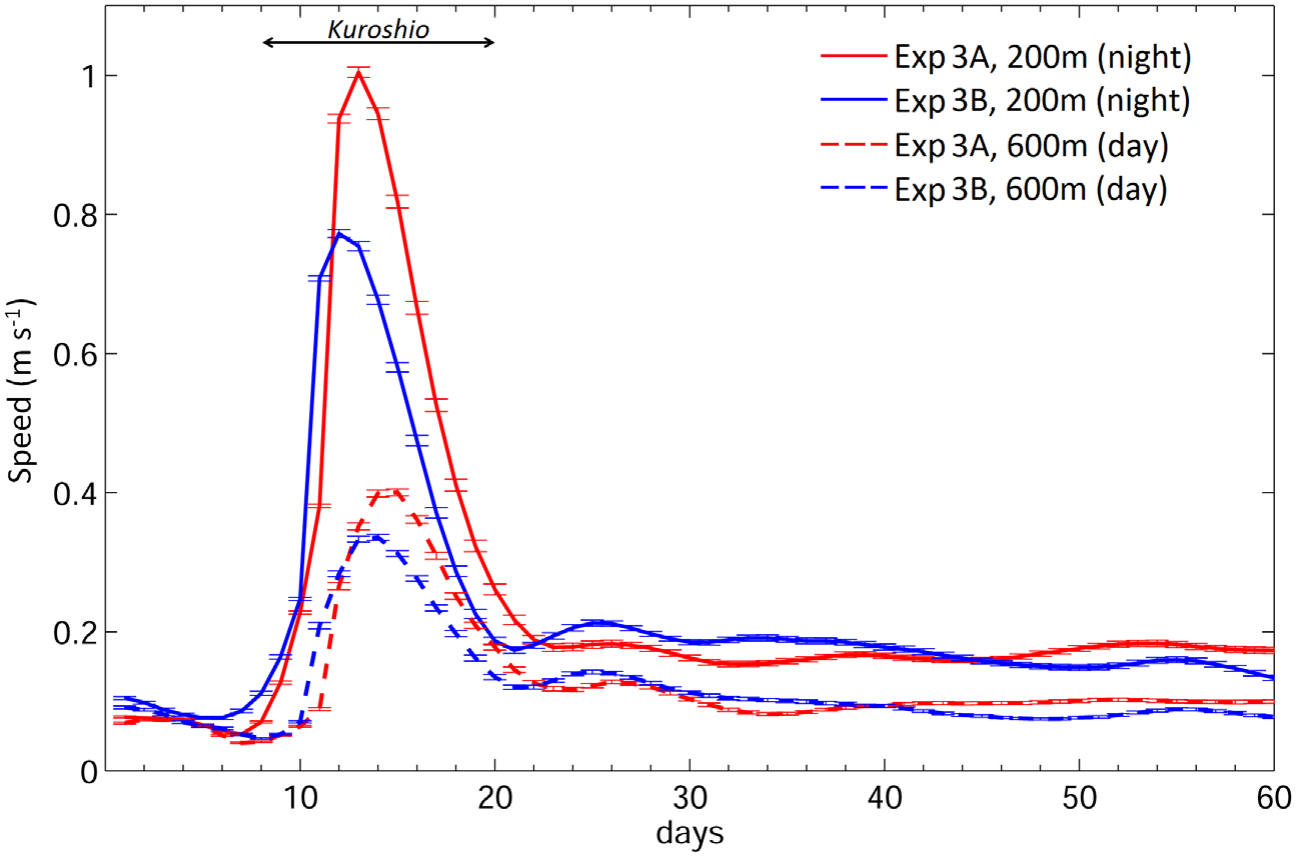
Mean along-path ocean speeds during (dash) daytime and (solid) nighttime for (red) Exp. 3A and (blue) Exp. 3B.

**Fig 8:**
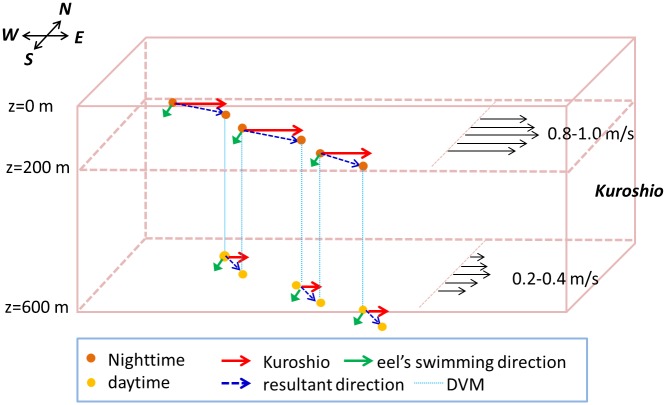
Schematic diagram showing how a v-eel swims across the Kuroshio. Black arrows represent the Kuroshio condition. During nighttime, the v-eel stays near the surface. The eastward strong Kuroshio together with the southward swimming direction of the v-eel leads to an east-south-east movement. The v-eel dives to deeper water during the daytime, where the weaker (than at the surface) Kuroshio and southward swimming results in a southeast migration. A few days later, the v-eel is able to cross the Kuroshio because of the accumulation of southward swimming.

Truly navigating v-eels could swim across the Kuroshio if they swam faster than 0.1 m s^−1.^ The composite along-path ocean current speeds from the standard case (Exp. 3) showed that v-eels took 12–14 days to move across the Kuroshio, which flowed eastward at a speed of 0.2–1.0 m s^−1^ (Fig 7). The manner in which the truly navigating v-eels (i.e., Exp. 3 and Exp. 6) swam across the Kuroshio was schematically explained in Fig 8. The Kuroshio speed decreases with depth, and v-eels stay in shallower waters during nighttime. The faster eastward Kuroshio and the slower southward swimming speed of v-eels result in east–southeast movement. V-eels drift further eastward at night because of the Kuroshio. During daytime, v-eels dive to a deeper layer, where the Kuroshio speed is weaker. The comparable eastward Kuroshio speed and the southward swimming v-eel speed lead to a southeast migration direction. V-eels are able to move further south in the deeper water during daytime. The cycle is repeated diurnally. The gradual southeast movement allows v-eels to ultimately swim across the Kuroshio.

To assess the role of ocean current on eel migration, Exp. 3 (v-eels truly navigating at 0.15 m s^−1^) was repeated by excluding background ocean circulation. V-eels in Exp. 3 were affected by the ocean current, showing winding paths and widely distributed trajectories (Figs 3 & 4). With ocean circulation, the average migration distance of v-eels in Exp. 3 was 2600–2800 km, whereas in the absence of ocean currents, the migration distance was reduced by 10% to 15% (2350 km). One may expect a shorter migration duration in the case without ocean currents because the v-eels swim at the same speed for a shorter migration distance. However, the migration duration of v-eels in the absence of ocean currents was longer than that in the presence of currents (Fig 5). The longer distance and shorter migration duration in Exp. 3 suggested that ocean currents accelerate the oceanic migration of eels.

All experiments were conducted under two extreme Kuroshio conditions to assess their effect on v-eel migration. In the case of a strong Kuroshio (Exp. A), v-eels were advected further eastward than in the weak Kuroshio case (Exp. B). The arrival rates were almost the same for both strong and weak Kuroshio conditions because of the long duration of the hatching season, although the migration durations differed under the different ocean current conditions (Table 2). V-eels swimming against the Kuroshio exhibited a significantly longer migration time (~5 days, p < 0.001) in the strong Kuroshio case than in the weak Kuroshio case (Exp. 8). The stronger Kuroshio counteracted the swimming efficiency of the v-eels, resulting in a lower moving speed and longer migration duration than those under the weak Kuroshio condition. V-eels truly navigating at 0.15 m s^−1^ exhibited a shorter migration duration (by 10 days) under the strong Kuroshio condition (Exp. 3). However, the time taken to swim across the Kuroshio was similar in both cases. Under a strong Kuroshio condition, the v-eels took 1–2 days longer to move over the jet. The migration duration difference suggested that the effects of both the Kuroshio and the ocean circulation south of the Kuroshio need to be considered in relation to eel migration.

**Fig 9:**
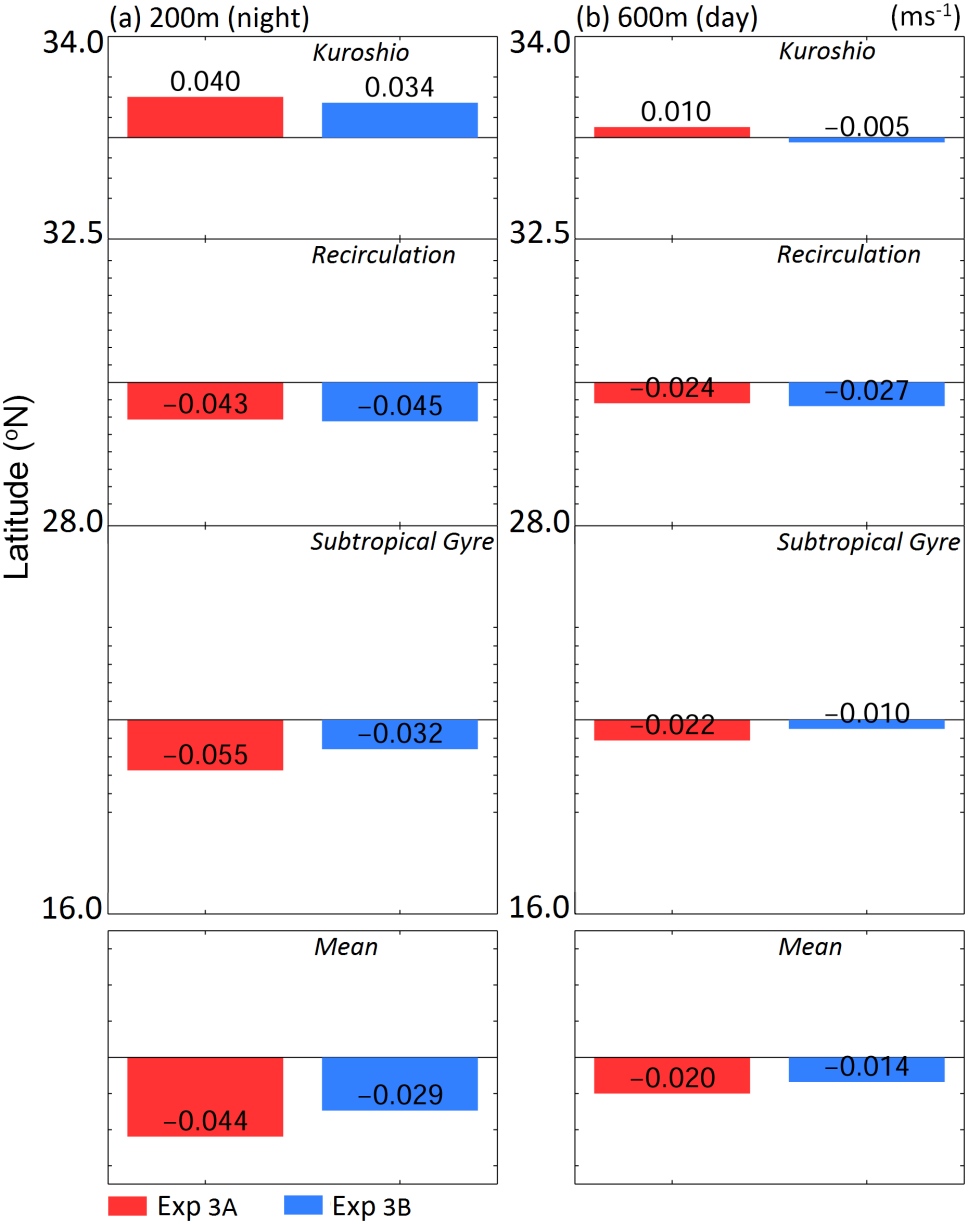
Along-path meridional velocity (m s^−1^) in the Kuroshio, recirculation region, subtropical gyre, and mean velocity for (a) nighttime and (b) daytime in Exp. 3.

The Kuroshio is a distinct feature on the eel migration route. The circulation south of the Kuroshio was weaker than the Kuroshio, but accounted for more than 90% of the migration distance; hence, its influence on eel migration was not negligible. For truly navigating v-eels, which mainly swam southward, zonal velocity led to zonal excursions of the eels (Fig 3) and meridional velocity accelerated or decelerated their migration speed. In Fig 9, the along-path meridional velocity was used to show the effect of ocean currents on the migration duration. In Exp. 3A (red bars in Fig 9), a slightly stronger northward (anti-migration direction) Kuroshio caused v-eels to take 1–2 days longer to swim across the Kuroshio than they did in Exp. 3B (blue bars in Fig 9). In addition, a stronger southward velocity (along-migration direction) in the subtropical gyre region in Exp. 3A accelerated v-eels so that they arrived at the spawning area earlier than they did in Exp. 3B. To confirm that the ocean condition can influence migration duration, we conducted an additional experiment with a stronger mean southward velocity (−0.059 m s^−1^) than that of Exp. 3A (−0.044 m s^−1^, Fig 9). The migration duration was thus shortened to 150.6 days, which is ~6 days faster than that for Exp. 3A. This result confirmed that a stronger southward ocean current can accelerate v-eel migration. Similarly, the shorter migration duration in the standard case (when compared with the case without ocean currents) is due to the contribution of southward (along-migration route) currents.

## Discussion

In the present study, we simulated the possible oceanic migration routes of Japanese silver eels over the western North Pacific Ocean using a 3D particle-tracking method, in which v-eels were programmed to move according to various swimming behaviors. V-eels released off Japan could reach the spawning area located 2300 km away in time to breed by continuously and actively swimming at biologically realistic minimal speeds following a readjusted heading (true navigation) or a constant heading (compass orientation) or by swimming against the current. The minimum swimming speed required for v-eels to reach the spawning area before the end of the breeding season (critical swimming speed) was the lowest for the case of true navigation (0.1 m s^−1^) and highest for the case of swimming against the Kuroshio (0.35 m s^−1^). In the case of compass orientation, the critical swimming speed was similar to that in the case of true navigation (0.12 m s^-1^), but the success rate was lower (60% vs. >90%). Indeed, some of the v-eels swimming in a constant direction (i.e., without readjusted heading) were deflected eastward and never entered the spawning area in the absence of behavior modification.

Our numerical experiments showed that the lowest horizontal swimming speeds observed in Japanese silver eels tracked at sea by Manabe et al. [19] would not allow v-eels to reach the spawning area in time to breed, even in the case of the most successful behavior (true navigation). However, the slow observed swimming speeds may be attributed to the negative effect of carrying a satellite tag, which may significantly impair the swimming performance of the eels [33]. At the assumed optimal swimming speed of 0.65 m s^−1^, truly navigating v-eels reached the spawning area eight weeks before the beginning of the spawning period, whereas v-eels taking the against-Kuroshio route arrived at the beginning of the spawning period. However, this optimal swimming speed was assessed in laboratory experiments for European eels at a constant temperature (18°C) [32]; the actual swimming speed of Japanese eels in the wild could be different.

The true navigation behavior appears very likely, although no definitive evidence can be presented so far. Such a mechanism may rely on the ability of eels to utilize the Earth’s magnetic field (similar to Pacific salmon [17]), given that eels are able to sense environmental magnetic fields and possess a magnetic compass [34,35]. Recently, a silver American eel was tracked from the Canadian coast to the northern spawning area in the Sargasso Sea [36], and the directionality and speed of this eel tracked in the open ocean suggest that eels have true navigation abilities. Although the true navigation strategy results in a high arrival rate and a lower minimum required swimming speed, it may not be the most efficient way because v-eels readjust their direction only considering the final destination without taking into account the energy cost related to the environmental conditions.

Laboratory experiments showed that eels can swim against the current [18] and that they display a negative rheotaxis in both welt water and natural stream water [37]. However, such an orientation strategy is relatively uncommon in long-distance migration because the energy cost of sustained movement into the flow would be too high [38]. The migration route against the Kuroshio could be energetically costly, and this migratory route may thus be unrealistic. In future studies, it will be interesting to test this hypothesis by calculating the energy expenditure along the migratory paths.

The present study shows that ocean circulation significantly affects the migration of Japanese v-eels. The strong Kuroshio could advect the v-eels further eastward, and the western Pacific Ocean currents accelerated the oceanic migration because of the contribution of a southward (along-migration path) current. The migration duration was shortened when the southward current was strengthened, which could be caused by a stronger recirculation south of Japan, an enhanced subtropical gyre, or a Kuroshio with a relatively large southward velocity. The Kuroshio south of Japan could take different paths: the “straight path” seen in the present study (Fig 2) and the offshore “large meander path” [39]. The offshore and looping Kuroshio in the large meander state may influence oceanic eel migration. The detailed effect of the large meander on oceanic migration is a topic for future studies. Ongoing climate change has remarkable effects on atmospheric and oceanic conditions [40]. Variations in ocean currents positively affect eel migration and may therefore further influence their breeding and future population structure.

The present study examined the effect of extreme Kuroshio conditions on the migration of Japanese silver eels. Oceanic conditions are highly time dependent and involve many different factors such as eddies, the Kuroshio large meander, the recirculation, and the subtropical gyre. It would be interesting to conduct experiments in the future using data for various years to identify the effect of the individual factors on the eel migration and to obtain more general conclusions.

## References

[1] Aida K, Tsukamoto K, Yamauchi K (2003) Eel Biology. Springer Japan: 497.

[2] Jacoby D, Gollock M (2014) Anguilla japonica The IUCN Red List of Threatened Species Version 2015–3.

[3] Tesch F (2003) The eel. Blackwell publishing: 408.

[4] Dekker W (2003) Did lack of spawners cause the collapse of the European eel, Anguilla anguilla? Fisheries Management and Ecology 10: 365–376.

[5] Baltazar-Soares M, Biastoch A, Harrod C, Hanel R, Marohn L, Prigge E, et al (2014) Recruitment collapse and population structure of the European eel shaped by local ocean current dynamics. Curr Biol 24: 104–108. 10.1016/j.cub.2013.11.031 24374306

[6] Bonhommeau S, Chassot E, Planque B, Rivot E, Knap A, Le Pape O (2008) Impact of climate on eel populations of the Northern Hemisphere. Marine Ecology Progress Series 373: 71–80.

[7] Knights B (2003) A review of the possible impacts of long-term oceanic and climate changes and fishing mortality on recruitment of anguillid eels of the Northern Hemisphere. Science of The Total Environment 310: 237–244. 12812748 10.1016/S0048-9697(02)00644-7

[8] Tsukamoto K (2009) Oceanic migration and spawning of anguillid eels. J Fish Biol 74: 1833–1852. 10.1111/j.1095-8649.2009.02242.x 20735675

[9] Okamura A, Yamada Y, Tanaka S, Horie N, Utoh T, Mikawa N, et al (2002) Atmospheric depression as the final trigger for the seaward migration of the Japanese eel Anguilla japonica. Marine Ecology Progress Series 234: 281–288.

[10] Tsukamoto K (2006) Oceanic biology: Spawning of eels near a seamount. Nature 439: 929–929. 16495988 10.1038/439929a

[11] Aoyama J, Watanabe S, Miller MJ, Mochioka N, Otake T, Yoshinaga T, et al (2014) Spawning Sites of the Japanese Eel in Relation to Oceanographic Structure and the West Mariana Ridge. PLoS ONE 9: e88759 10.1371/journal.pone.0088759 24551155 PMC3923831

[12] Shinoda A, Aoyama J, Miller M, Otake T, Mochioka N, Watanabe S, et al (2011) Evaluation of the larval distribution and migration of the Japanese eel in the western North Pacific. Reviews in Fish Biology and Fisheries 21: 591–611.

[13] Kida S, Mitsudera H, Aoki S, Guo X, Ito S-i, Kobashi F, et al (2015) Oceanic fronts and jets around Japan: a review. Journal of Oceanography: 1–29.

[14] Nagano A, Ichikawa K, Ichikawa H, Konda M, Murakami K (2013) Volume transports proceeding to the Kuroshio Extension region and recirculating in the Shikoku Basin. Journal of Oceanography 69: 285–293.

[15] Tsukamoto K (1994) Origin of diadromous fishes and mechanism of migration. In Freshwater Fishes Migrating Between River and the Sea: 2–17.

[16] Yokose H (2008) Geological approach to the spawning sites of the Japanese eel. Kaiyo Monthly Special Issue 48: 45–58.

[17] Putman Nathan F, Scanlan Michelle M, Billman Eric J, O’Neil Joseph P, Couture Ryan B, Quinn Thomas P, et al (2014) An Inherited Magnetic Map Guides Ocean Navigation in Juvenile Pacific Salmon. Current Biology 24: 446–450. 10.1016/j.cub.2014.01.017 24508165

[18] Russon IJ, Kemp PS (2011) Experimental quantification of the swimming performance and behaviour of spawning run river lamprey Lampetra fluviatilis and European eel Anguilla anguilla. J Fish Biol 78: 1965–1975. 10.1111/j.1095-8649.2011.02965.x 21651544

[19] Manabe R, Aoyama J, Watanabe K, Kawai M, Miller MJ, Tsukamoto K (2011) First observations of the oceanic migration of Japanese eel, from pop-up archival transmitting tags. Marine Ecology Progress Series 437: 229–240.

[20] Chow S, Okazaki M, Watanabe T, Segawa K, Yamamoto T, Kurogi H, et al (2015) Light-Sensitive Vertical Migration of the Japanese Eel *Anguilla japonica* Revealed by Real-Time Tracking and Its Utilization for Geolocation. PLoS ONE 10: e0121801 10.1371/journal.pone.0121801 25875179 PMC4398447

[21] Aarestrup K, Økland F, Hansen MM, Righton D, Gargan P, Castonguay M, et al (2009) Oceanic Spawning Migration of the European Eel (Anguilla anguilla). Science 325: 1660 10.1126/science.1178120 19779192

[22] Béguer-Pon M, Benchetrit J, Castonguay M, Aarestrup K, Campana SE, Stokesbury MJW, et al (2012) Shark Predation on Migrating Adult American Eels (*Anguilla rostrata*) in the Gulf of St. Lawrence. PLoS ONE 7: e46830 10.1371/journal.pone.0046830 23082131 PMC3474790

[23] Gentleman W (2002) A chronology of plankton dynamics in silico: how computer models have been used to study marine ecosystems. Hydrobiologia 480: 69–85.

[24] Miyazawa Y, Zhang R, Guo X, Tamura H, Ambe D, Lee J-S, et al (2009) Water mass variability in the western North Pacific detected in a 15-year eddy resolving ocean reanalysis. Journal of Oceanography 65: 737–756.

[25] Mellor G, Häkkinen S, Ezer T, Patchen R (2002) A Generalization of a Sigma Coordinate Ocean Model and an Intercomparison of Model Vertical Grids. In Ocean Forecasting: Conceptual Basis and Applications: 55–72.

[26] Chang Y-L, Sheng J, Ohashi K, Béguer-Pon M, Miyazawa Y (2015) Impacts of Interannual Ocean Circulation Variability on Japanese Eel Larval Migration in the Western North Pacific Ocean. PLoS ONE 10: e0144423 10.1371/journal.pone.0144423 26642318 PMC4671650

[27] Ohashi K, Sheng J (2015) Investigating the Effect of Oceanographic Conditions and Swimming Behaviours on the Movement of Particles in the Gulf of St. Lawrence Using an Individual-Based Numerical Model. Atmosphere-Ocean: 1–21.

[28] Press W, Teukolsky S, Vetterling W, Flannery B (1992) Numerical recipes in Fortran 77: the art of scientific computing. Cambridge University Press.

[29] Shan S, Sheng J, Greenan B (2014) Modelling study of three-dimensional circulation and particle movement over the Sable Gully of Nova Scotia. Ocean Dynamics 64: 117–142.

[30] Béguer-Pon M, Shan S, Thompson KR, Castonguay M, Sheng J, Dodson JJ (2015) Exploring the role of the physical marine environment in silver eel migrations using a biophysical particle tracking model. ICES Journal of Marine Science: Journal du Conseil.

[31] Rypina II, Llopiz JK, Pratt LJ, Susan Lozier M (2014) Dispersal pathways of American eel larvae from the Sargasso Sea. Limnology and Oceanography 59: 1704–1714.

[32] Palstra A, van Ginneken V, van den Thillart G (2008) Cost of transport and optimal swimming speed in farmed and wild European silver eels (Anguilla anguilla). Comp Biochem Physiol A Mol Integr Physiol 151: 37–44. 10.1016/j.cbpa.2008.05.011 18599333

[33] Methling C, Tudorache C, Skov PV, Steffensen JF (2011) Pop Up Satellite Tags Impair Swimming Performance and Energetics of the European Eel (*Anguilla anguilla*). PLoS ONE 6: e20797 10.1371/journal.pone.0020797 21687674 PMC3110781

[34] Durif CMF, Browman HI, Phillips JB, Skiftesvik AB, Vøllestad LA, Stockhausen HH (2013) Magnetic Compass Orientation in the European Eel. PLoS ONE 8: e59212 10.1371/journal.pone.0059212 23554997 PMC3598651

[35] Tesch FW (1974) Influence of geomagnetism and salinity on the directional choice of eels. Helgoländer wissenschaftliche Meeresuntersuchungen 26: 382–395.

[36] Beguer-Pon M, Castonguay M, Shan S, Benchetrit J, Dodson JJ (2015) Direct observations of American eels migrating across the continental shelf to the Sargasso Sea. Nat Commun 6. 10.1038/ncomms9705 PMC4918406 26505325

[37] Hain JHW (1975) The behaviour of migratory eels,Anguilla rostrata, in response to current, salinity and lunar period. Helgoländer wissenschaftliche Meeresuntersuchungen 27: 211–233.

[38] Chapman Jason W, Klaassen Raymond HG, Drake VA, Fossette S, Hays Graeme C, Metcalfe Julian D, et al (2011) Animal Orientation Strategies for Movement in Flows. Current Biology 21: R861–R870. 10.1016/j.cub.2011.08.014 22032194

[39] Kawabe M (1995) Variations of Current Path, Velocity, and Volume Transport of the Kuroshio in Relation with the Large Meander. Journal of Physical Oceanography 25: 3103–3117.

[40] Ipcc (2014) Climate Change 2014: Impacts, Adaptation, and Vulnerability. Part A: Global and Sectoral Aspects. Contribution of Working Group II to the Fifth Assessment Report of the Intergovernmental Panel on Climate Change [Field, C.B., V.R. Barros, D.J. Dokken, K.J. Mach, M.D. Mastrandrea, T.E. Bilir, M. Chatterjee, K.L. Ebi, Y.O. Estrada, R.C. Genova, B. Girma, E.S. Kissel, A.N. Levy, S. MacCracken, P.R. Mastrandrea, and L.L. White (eds.)]. Cambridge, United Kingdom and New York, NY, USA: Cambridge University Press. 1132 p.

